# Nuclear matrix protein Matrin 3 is a regulator of ZAP-mediated retroviral restriction

**DOI:** 10.1186/s12977-015-0182-4

**Published:** 2015-07-01

**Authors:** Angela Erazo, Stephen P Goff

**Affiliations:** Department of Biochemistry and Molecular Biophysics, Howard Hughes Medical Institute, Columbia University, New York, NY 10032 USA; Department of Microbiology and Immunology, Columbia University, New York, NY 10032 USA

**Keywords:** Nuclear matrix, Retrovirus, ZAP, Matrin 3, Restriction factor, RNA degradation, ZC3HAV1, HIV-1

## Abstract

**Background:**

Matrin 3 is a nuclear matrix protein involved in multiple nuclear processes. In HIV-1 infection, Matrin 3 serves as a Rev cofactor important for the cytoplasmic accumulation of HIV-1 transcripts. ZAP is a potent host restriction factor of multiple viruses including retroviruses HIV-1 and MoMuLV. In this study we sought to further characterize Matrin 3 functions in the regulation of HIV gene expression.

**Results:**

Here we describe a function for Matrin 3 as a negative regulator of the ZAP-mediated restriction of retroviruses. Mass spectrometry analysis of Matrin 3-associated proteins uncovered interactions with proteins of the ZAP degradation complex, DDX17 and EXOSC3. Coimmunoprecipitation studies confirmed Matrin 3 associations with DDX17, EXOSC3 and ZAP, in a largely RNA-dependent manner, indicating that RNA is mediating the Matrin 3 interactions with these components of the ZAP degradation complex. Silencing Matrin 3 expression caused a remarkably enhanced ZAP-driven inhibition of HIV-1 and MoMuLV luciferase reporter viruses. This effect was shared with additional nuclear matrix proteins. ZAP targets multiply-spliced HIV-1 transcripts, but in the context of Matrin 3 suppression, this ZAP restriction was broadened to unspliced and multiply-spliced RNAs.

**Conclusions:**

Here we reveal an unprecedented role for a nuclear matrix protein, Matrin 3, in the regulation of ZAP’s antiretroviral activity. Suppressing Matrin 3 powers a heightened and broader ZAP restriction of HIV-1 gene expression. This study suggests that this ZAP regulatory mechanism is shared with additional nuclear matrix proteins.

## Background

Matrin 3 was first described as a major nuclear matrix protein and since has been associated with multiple nuclear processes [1]. The nuclear matrix is a proteinaceous structure that provides a dynamic spatial organization to the mammalian nucleus [2]. This matrix is defined as the nonhistone subnuclear fraction that is resistant to stringent biochemical procedures including high salt, detergent and nuclease treatment [3, 4] with a large proportion comprised of RNA-binding proteins [5]. Matrin 3 is a highly conserved 125 kDa inner matrix protein containing two RNA recognition motifs (RRM) that bind RNA [6, 7] and two zinc fingers that are predicted to bind DNA, displaying a diffuse predominantly nuclear localization and nucleolar exclusion [1, 8, 9]. Matrin 3 has been involved in a variety of functions, such as mediating neuronal cell death through its phosphorylation by PKA following NMDA receptor activation [10], and its role as a component of a p54nrb-PSF complex that anchors hyperedited nuclear dsRNAs to the nucleus to prevent the erroneous translation of potentially nonfunctional mutant proteins [11]. In complex with p54nrb-PSF, Matrin 3 has also been linked to early stages of the DNA double strand break response [12]. Matrin 3 has been implicated in DNA replication/repair, translation [9], chromatin organization [9, 13], and RNA stability and processing [7, 9], often through its interaction with proteins functioning in these processes. Additionally, Matrin 3 was also found to be an integral component of a dynamic scaffold aiding the organization of “nuclear functional neighborhoods” where proteins involved in chromatin replication and transcription are concentrated [14]. Clinically Matrin 3 has been linked as a cause of the neurodegenerative disease familial amyotrophic lateral sclerosis (ALS), interacting with the RNA binding protein TDP-43, a protein that when mutated is known to cause ALS [15].

As a cellular protein target of viruses, Matrin 3 has been linked to both DNA and RNA viruses. Matrin 3 was initially described as a common target of multiple alphaherpesviruses through a specific phosphorylation driven by the conserved US3 viral kinase [8]. During HIV-1 infection, it was demonstrated that Matrin 3 binds HIV-1 RNAs [6] and acts as a Rev cofactor, promoting the accumulation of Rev-dependent unspliced and singly-spliced HIV-1 RNAs [6, 16] in the cytoplasm. HIV-1 encodes nine proteins through strictly controlled splicing of viral RNA resulting in unspliced (Gag, Gag-Pol), singly-spliced (Env, Vif, Vpr, Vpu), and multiply-spliced (Tat, Rev, Nef) transcripts. Controlled levels of these transcripts are crucial to efficient HIV-1 replication [17]. Moreover, Matrin 3’s RRMs are necessary for its Rev-dependent RNA stabilization and expression of Gag protein [16]. These studies pointed out that this effect on Gag was likely at a post-transcriptional step, as Matrin 3 levels did not seem to affect transcription [6, 16].

The host factor, CCCH-type Zinc-Finger Antiviral protein (ZAP) is a potent retroviral restriction factor that was originally discovered on the basis of its activity against Moloney MuLV (murine leukemia virus), and leads to the striking degradation of viral RNAs specifically in the cytoplasm [18]. Later studies revealed ZAP’s function as a restriction factor against many viruses including multiple alphaviruses [19], the filoviruses Ebola and Marburg viruses [20], hepatitis B virus [21], and its ability to regulate murine gammaherpesvirus 68 latency [22]. ZAP does not act as a universal antiviral factor as some viruses such as herpes simplex virus type 1 and poliovirus were not affected by its expression [19]. Using MuLV vectors, ZAP was found to act through binding to viral RNAs [23] and recruiting the RNA processing exosome, a 3–5′ exoribonuclease complex [24] and p72 DEAD box RNA helicase (DDX17) for optimal RNA degradation [25, 26]. ZAP also restricts replication of HIV-1 [27]. Further studies demonstrated that ZAP activity is directed principally to multiply-spliced HIV-1 RNAs through the utilization of 3′–5′ RNA degradation machinery, while also recruiting components of the cellular decapping complex through p72 to degrade RNA through 5′–3′ exoribonuclease Xrn1 [27]. Furthermore it was found that ZAP induces translational repression through its interaction with eIF4A, and this disruption of translation initiation is required for degradation of HIV-1 RNAs to occur [28].

In this study we find an unexpected role for Matrin 3 as a regulator of the antiviral activity of ZAP. We demonstrate that Matrin 3 interacts with key components of the RNA degradation machinery, and that suppression of Matrin 3 expression greatly enhances ZAP antiviral activity against HIV-1 RNAs. Importantly, loss of Matrin 3 in conjunction with ZAP expression broadens the restrictive activity to drive the degradation of both unspliced and multiply spliced HIV-1 transcripts. These result suggest that Matrin 3 is a negative regulator of ZAP.

## Results

### Matrin 3 interacts with components of the ZAP degradation machinery in an RNA-dependent manner

To explore the role of Matrin 3 in HIV-1 replication, we screened for Matrin 3 interacting proteins in both HIV-1-infected and uninfected cells. Cell lysates were prepared from uninfected human brain microglial CHME3 [29], or CHME3 infected with a VSV-G pseudotyped HIV-1 vector pnl4.3env^-^luc^+^ (HIV-luc) using mild detergent conditions. CHME3 cells were used in these experiments as being highly permissive for HIV-1 replication and representative of microglial cells naturally infected by HIV-1 [30]. Endogenous Matrin 3 was then recovered by immunoprecipitation and bound proteins were identified by mass spectrometric analysis. Many Matrin 3-interacting proteins were identified in both uninfected and infected cells (listed in Table 1a), and a number of proteins were identified only in HIV-luc infected CHME3 (Table 1b). DDX17 and EXOSC3 were noteworthy as known components of the RNA degradation machinery recruited by the retroviral restriction factor, ZAP, appearing only in infected CHME3 cells. DDX17 is a cofactor of ZAP and an RNA helicase needed for optimal ZAP-mediated degradation of RNA, possibly through the restructuring of the secondary structure of ZAP-responsive mRNA elements [26]. DDX17 encodes two isoforms: p82 and a faster migrating p72 resulting from the use of different in-frame translation initiation codons. EXOSC3 is a core component of the human exosome complex responsible for 3’-5’ exoribonuclease activity [31].

**Table 1:** Proteins identified as Matrin 3 interacting proteins by mass spectrometry

Name	Synonyms	Accession #
(a) Identified as Matrin 3 interacting proteins in uninfected and infected cells		
Matrin 3*	MATR3, KIAA0723	P43243
Probable ATP-dependent RNA helicase DDX5	DDX5, G17P1, HELR, HLR1	P17844
Heterogeneous nuclear ribonucleoproteins A2/B1*	ROA2, HNRNPA2B1	P22626
ATP-dependent RNA helicase DDX3X	DDX3X, DDX3, DBX,	O00571
Heterogeneous nuclear ribonucleoprotein U	HNRPU, HNRNPU SAFA, U21.1	Q00839
Polypyrimidine tract-binding protein 1	PTBP1, PTB	P26599
Heterogeneous nuclear ribonucleoproteins C1/C2*	HNRPC, HNRNPC	P07910
Heterogeneous nuclear ribonucleoprotein M	HNRPM, HNRNPM, NAGR1	P52272
Heterogeneous nuclear ribonucleoprotein A1	ROA1, HNRPA1, HNRNPA1	P09651
(b) Identified as a Matrin 3 interacting protein in infected cells alone		
Probable ATP-dependent RNA helicase DDX17*	DDX17, RNA-dependent helicase p72	Q92841
ATP-dependent DNA helicase	RECQ1, RECQL1	P46063
Tubulin alpha-1A	TBA1A, TUBA3	Q71U36
Tubulin beta-2C chain	TBB2C, TUBB2C, TUBB4B	P68371
Cellular tumor antigen p53	P53, TP53	P04637
Heterogeneous nuclear ribonucleoprotein R	HNRPR, HNRNPR	O43390
Histone H1.2	H12, HIST1H1C, H1F2	P16403
Plasminogen activator inhibitor 1 RNA-binding protein	PAIRB, PAIRBP1, SERBP1	Q8NC51
Heterogeneous nuclear ribonucleoprotein K	HNRPK, HNRNPK	P61978
Tubulin beta-6 chain	TBB6, TUBB6	Q9BUF5
Tropomyosin alpha-1 chain	TPM1, C15orf13, TMSA	P09493
60S ribosomal protein L7	RL7, RPL7	P18124
Exosome complex component RRP40	EXOS3, EXOSC3, RRP40	Q9NQT5
SAFB-like transcription modulator	SLTM, MET	Q9NWH9
60S ribosomal protein L15	RL15, EC45	P61313
ELAV-like protein 1	ELAV1, ELAVL1, HUR	Q15717
60S ribosomal protein L18	RL18, RPL18	Q07020
Heterogeneous nuclear ribonucleoprotein A3	ROA3, HNRNPA3, HNRPA3	P51991

**Figure 1:**
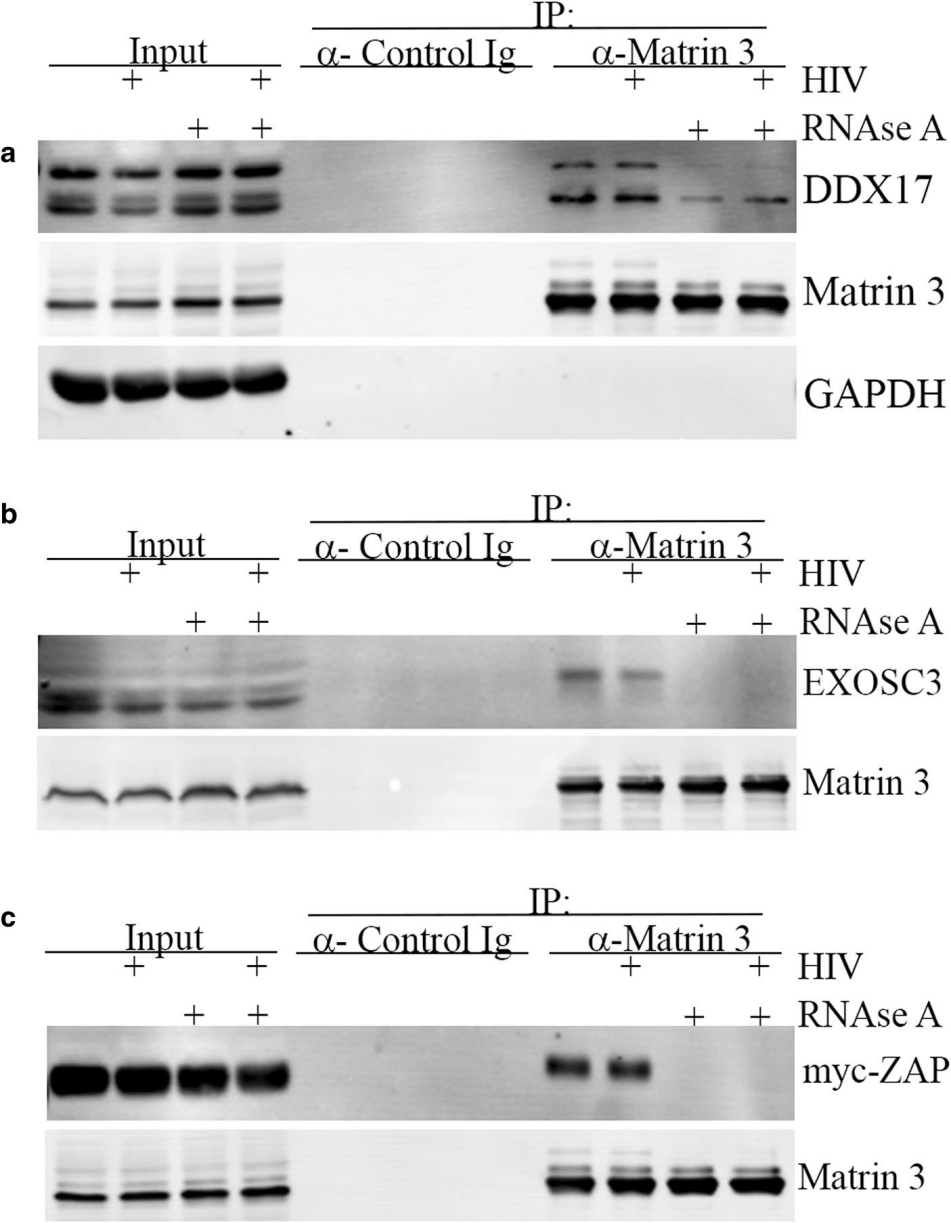
Matrin 3 interacts with components of the ZAP degradation machinery. **a** Matrin 3 interacts with DDX17. 293TrexhZAP2 cells were infected with HIV-luc (+) or mock infected (−) followed by doxycycline treatment at 200 ng/ml. Cells were then lysed in the presence (+) or absence (−) of RNAse A (50 µg/ml). Lysates were subjected to immunoprecipitation for endogenous Matrin 3 using rabbit α- Matrin 3 or α- control Ig. DDX17, GAPDH, **b** EXOSC3, or **c** myc-ZAP2 were detected with antibodies indicated.

To confirm that DDX17 and EXOSC3 interacted with Matrin 3, we tested for their coimmunoprecipitation with endogenous Matrin 3 in uninfected or infected 293TrexhZAP2 cells, a 293T cell line derivative in which doxycycline induces the expression of myc-tagged ZAP2, a functional human isoform truncated at the C-terminus. Additionally we tested for interaction with the central factor of this complex, ZAP. 293TrexhZAP2 cells were infected with a HIV-luc reporter virus and then treated with doxycycline at 2 h postinfection, and lysates were prepared 48 h after infection. Matrin 3 coimmunprecipitated with DDX17 with partial preference for the faster migrating p72 isoform, but not with the control GAPDH (Figure 1a). The association was partially disrupted by treatment with RNAse A. Matrin 3 also interacted with EXOSC3 (Figure 1b) and ZAP (Figure 1c) in a strongly RNA-dependent manner. Similar results were also obtained in TE671 cells (data not shown). This interaction of Matrin 3 with essential components of the RNA degradation machinery was observed in uninfected cells and with no apparent increase following HIV-luc infection. HIV-luc infection did not increase protein levels of DDX17, EXOSC3, or myc-ZAP2.

### Matrin 3 suppression enhances ZAP-mediated restriction of retroviral infection

**Figure 2:**
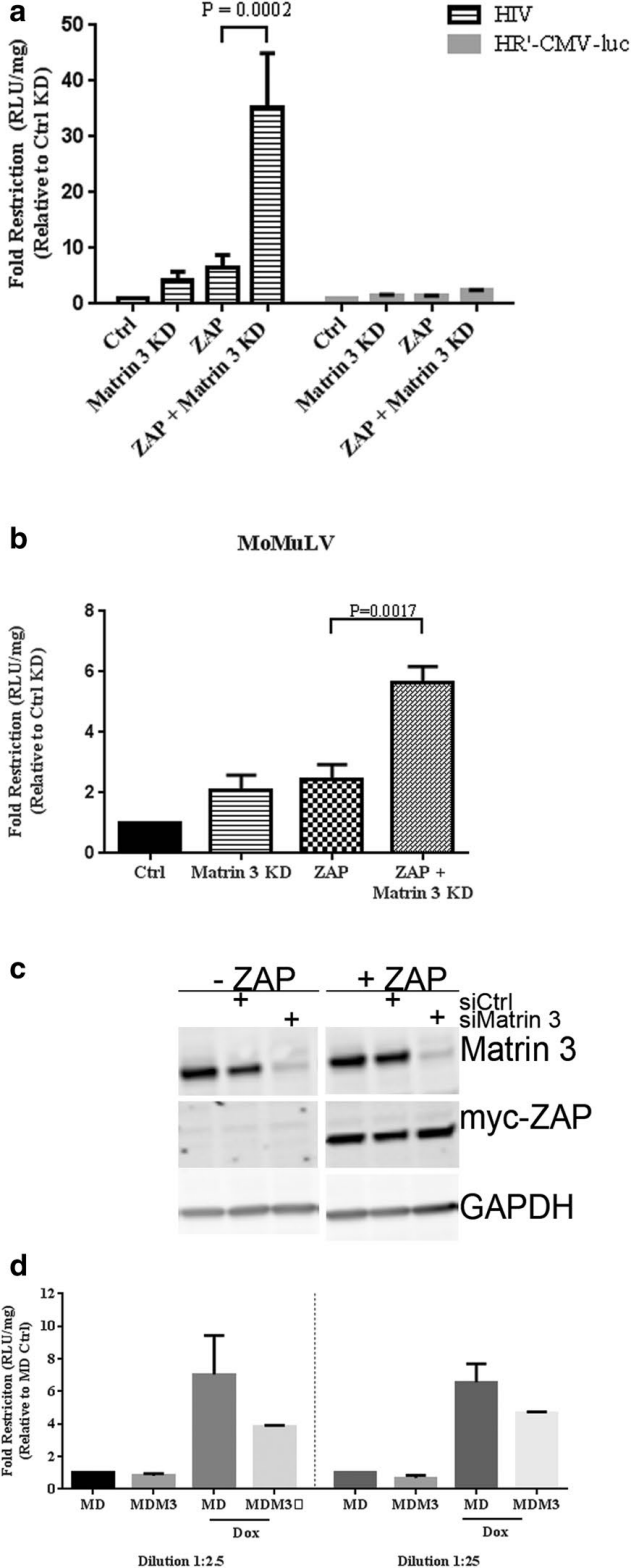
Matrin 3 suppression enhances ZAP-mediated restriction of retroviral infection. **a** Enhanced ZAP-mediated HIV-1 restriction in the absence of Matrin 3 expression. 293TrexhZAP2 cells (Ctrl) or cells silenced for Matrin 3 expression (Matrin 3 KD) were infected with HIV-luc or HR’-CMV-luc then treated with (ZAP) or without doxycycline treatment at 200 ng/ml. Cells were then harvested 2 days postinfection and lysates were subjected to luciferase assays to measure Nef-luc reporter activity. Data presented are the mean RLU/mg values of at least three experiments done in triplicate ±SD. Fold restriction was calculated from dividing Ctrl siRNA values by the results of each treatment indicated. **b** MoMuLV restriction also improved by Matrin 3 silencing. Data presented are the mean RLU/mg values of three independent experiments done in triplicate. P values ≤0.05 are shown. **c** Knockdown of Matrin 3 by siRNA treatment. Lysates from a representative experiment were subjected to immunoblot to illustrate Matrin 3 knockdown. Lysates were analyzed for at least three independent experiments. 293TrexhZAP2 were treated with control siRNA (siCtrl) or siRNA to Matrin 3 (siMatrin 3) for two consecutive days followed by infection and doxycycline treatment as stated above. Induction of myc-ZAP2 overexpression was detected with mouse α-myc antibody. GAPDH is the protein loading the control. **d** Cells transiently expressing mycDDK-tagged Matrin 3 (MDM3) or vector alone (MD) were infected with HIV-luc at two different dilutions, then treated with (ZAP) or without doxycycline (Dox) treatment at 200 ng/ml. Cells were then harvested as stated above. Data presented are the mean RLU/mg values of two experiments done in triplicate. Fold restriction was calculated from dividing MD values by the results of each treatment indicated for each dilution.

The observation that Matrin 3 was associated with multiple proteins essential to the restriction involved in degrading HIV-1 mRNAs raised the possibility of a functional involvement. To test if Matrin 3 had a role in the ZAP-mediated restriction of HIV-1, we overexpressed ZAP to restrict HIV-1 replication in HIV-luc infected 293TrexhZAP2 cells, and tested for ZAP restriction activity with and without knockdown of Matrin 3. We found that ZAP alone induced an approximately sixfold restriction of HIV-1 Nef-luc expression in these cells, and that Matrin 3 knockdown alone led to a fourfold inhibition of HIV-1 Nef-luc expression. Since this reporter is Rev-independent, this effect is likely separate from Matrin 3’s function as a Rev cofactor, but may reflect the broad role of Matrin 3 in affecting mRNA processing and expression. Remarkably, knockdown of Matrin 3 expression in the context of ZAP overexpression led to a profound increase in ZAP-mediated HIV-1 restriction, resulting in a 35-fold restriction (Figure 2a). HR’-CMV-luc has a large portion of the HIV genome deleted and has previously been shown to be insensitive to ZAP restriction [27]. Infection with HR’-CMV-luc showed very modest reductions under all conditions tested, demonstrating that Matrin 3 knockdown in the context of ZAP overexpression was not causing a global decrease of newly expressed luciferase reporter (Figure 2a). Similar experiments were performed with infection with HIV-1 at three dilutions of virus (1:2 to 1:200), and a 35-fold restriction on average was seen at all virus dilutions (data not shown). To determine if this finding was HIV-1-specific, we also tested for the effects of Matrin 3 suppression on ZAP-mediated inhibition of Moloney murine leukemia virus (MoMuLV) expression. We observed a less dramatic but significant enhancement of ZAP-driven retroviral restriction. Matrin 3 KD or ZAP overexpression individually caused a twofold inhibition of virus expression, while the combination of both together led to a sixfold inhibition (Figure 2b). Western blots showed that the knockdown of Matrin 3 and the overexpression of myc-ZAP2 were both achieved at high efficiency (Figure 2c). Conversely, we tested if overexpression of Matrin 3 would hinder ZAP activity. Myc-DDK tagged Matrin 3 was transiently expressed in 293TrexhZAP2 cells. Doxycycline was added to drive ZAP expression, the cells were then infected with HIV-Luc at two different dilutions, and lysates were collected 2 days postinfection. Exogenous overexpression of Matrin 3 decreased ZAP’s restrictive activity by approximately half at both dilutions (Figure 2d). Although not as striking as the effects on ZAPs activity during Matrin 3 suppression, the data are consistent with the concept that Matrin 3 impedes ZAP activity. Taken together, our data suggests that Matrin 3 is a negative regulator of ZAP-mediated restriction activity on retroviruses.

### Effects of other major nuclear matrix proteins on ZAP activity

**Figure 3:**
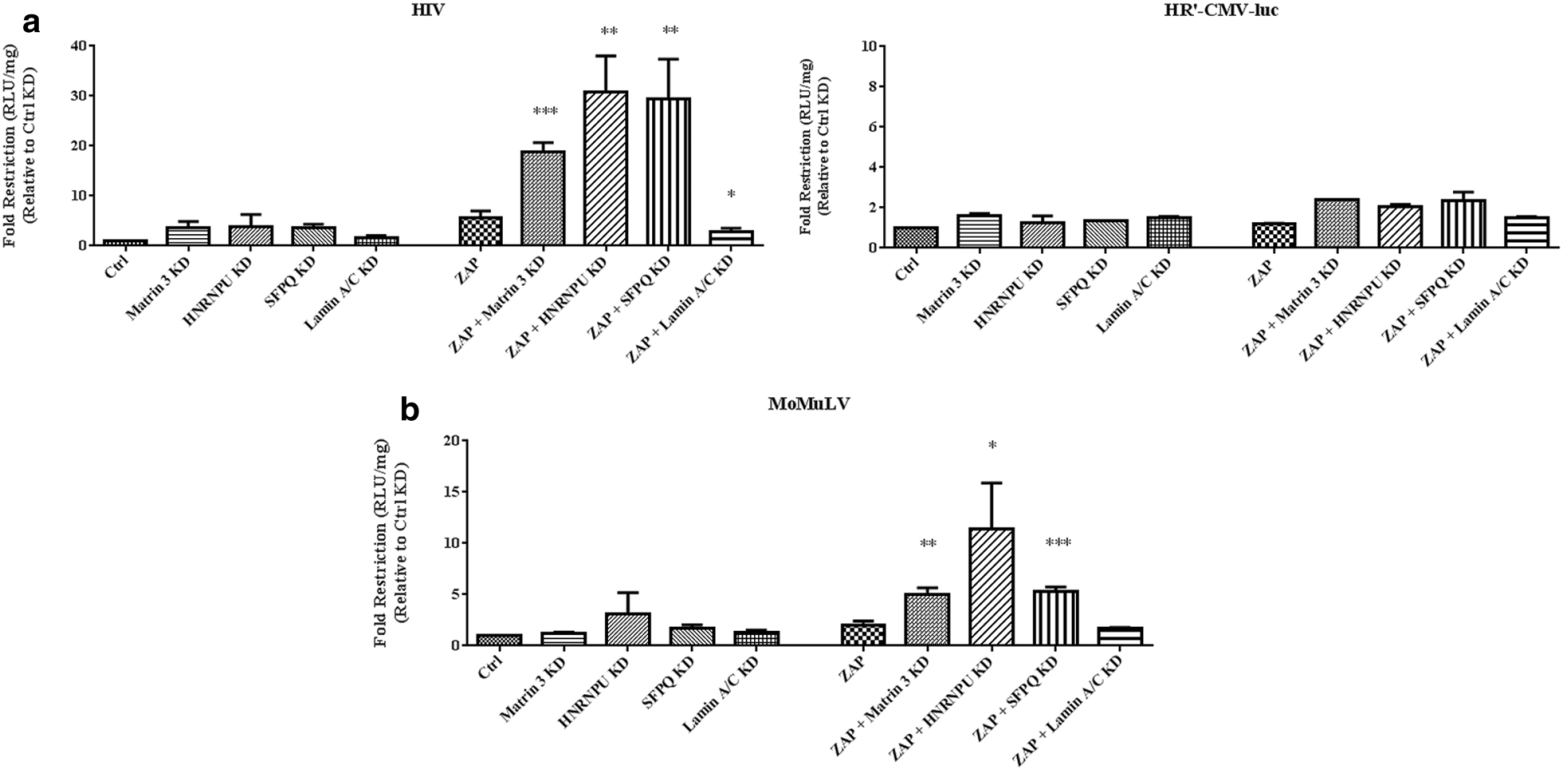
Effects of other major nuclear matrix proteins on zap activity. Distinct nuclear matrix proteins regulate ZAP effects on **a** HIV-1 or HR’-CMV-luc and **b** MoMuLV. 293TrexhZAP2 cells were transfected with control siRNA (Ctrl) or siRNA against Matrin 3 (Matrin 3 KD), HNRNPU (HNRNPU KD), SFPQ (SFPQ KD), or Lamin A/C (Lamin A/C KD) for two consecutive days. Subsequently on the following day, cells were infected with HIV-luc or HR’-CMV-luc then treated with (ZAP) or without doxycycline treatment at 200 ng/ml. Cells were then harvested 2 days postinfection and lysates were subjected to luciferase assays to measure Nef-luc reporter activity. Fold restriction was calculated from dividing Ctrl siRNA values by the results of each treatment indicated. Data presented are the mean RLU/mg values of three independent experiments done in triplicate ±SD (HIV and MoMuLV) and two experiments done in triplicate ±SD for HR’-CMV-luc. Statistical analyses were done to test for differences between ZAP and ZAP induced in cells treated with siRNA. P values ≤0.05 are shown.

Earlier studies indicated that Matrin 3 is one of a collection of major nuclear matrix proteins that create a nuclear scaffold affecting a diverse array of cellular functions including RNA processing. To explore the potential involvement of this group of matrix proteins on ZAP function, we tested the nuclear matrix proteins heterogeneous nuclear ribonucleoprotein U (HNRNPU), splicing factor proline/glutamine rich (polypyrimidine tract-binding protein-associated) (SFPQ), and lamin A/C for their effects on HIV-1 infection. These proteins have all been found as components of the nuclear matrix, an operationally defined fibrogranular nuclear subfraction [3–5, 32, 33]. HNRNPU [9, 34] and SFPQ [11, 12] are reported to form complexes with Matrin 3. Silencing of these nuclear proteins individually had modest effects on HIV-1 Nef-luc expression ranging from a 1.5-fold (for lamin A/C), up to a 3.9-fold effect (for HNRNPU) (Figure 3a). Notably, silencing of HNRNP U or SFPQ, but not lamin A/C, together with ZAP overexpression, led to enhanced ZAP restriction of HIV. While ZAP overexpression alone resulted in a 5.6-fold restriction, ZAP overexpression along with knockdown of HNRNPUKD or SFPQ increased the restriction to approximately 30-fold (Figure 3a). No significant increase in ZAP function was detected with knockdown of lamin A/C. Infection with HR’-CMV-luc showed very modest reductions under all conditions. This finding suggest a role for at least three nuclear matrix proteins in negatively regulating ZAP-mediated restriction of HIV-1 gene expression. A similar trend was noted in MoMuLV infection (Figure 3b). Silencing of matrix proteins alone showed only very subtle effects on restriction. ZAP overexpression alone caused a 2-fold restriction, while overexpression in Matrin 3 KD and SFPQ KD cells demonstrated a 5-fold restriction and overexpression in HNRNPU KD cells showed an 11-fold restriction. No such increase was seen in lamin A/C KD cells. These observations suggest that multiple nuclear matrix proteins can negatively regulate ZAP restriction of multiple viruses.

### Matrin 3 knockdown enhances the ZAP-induced degradation of HIV-1 transcripts

**Figure 4:**
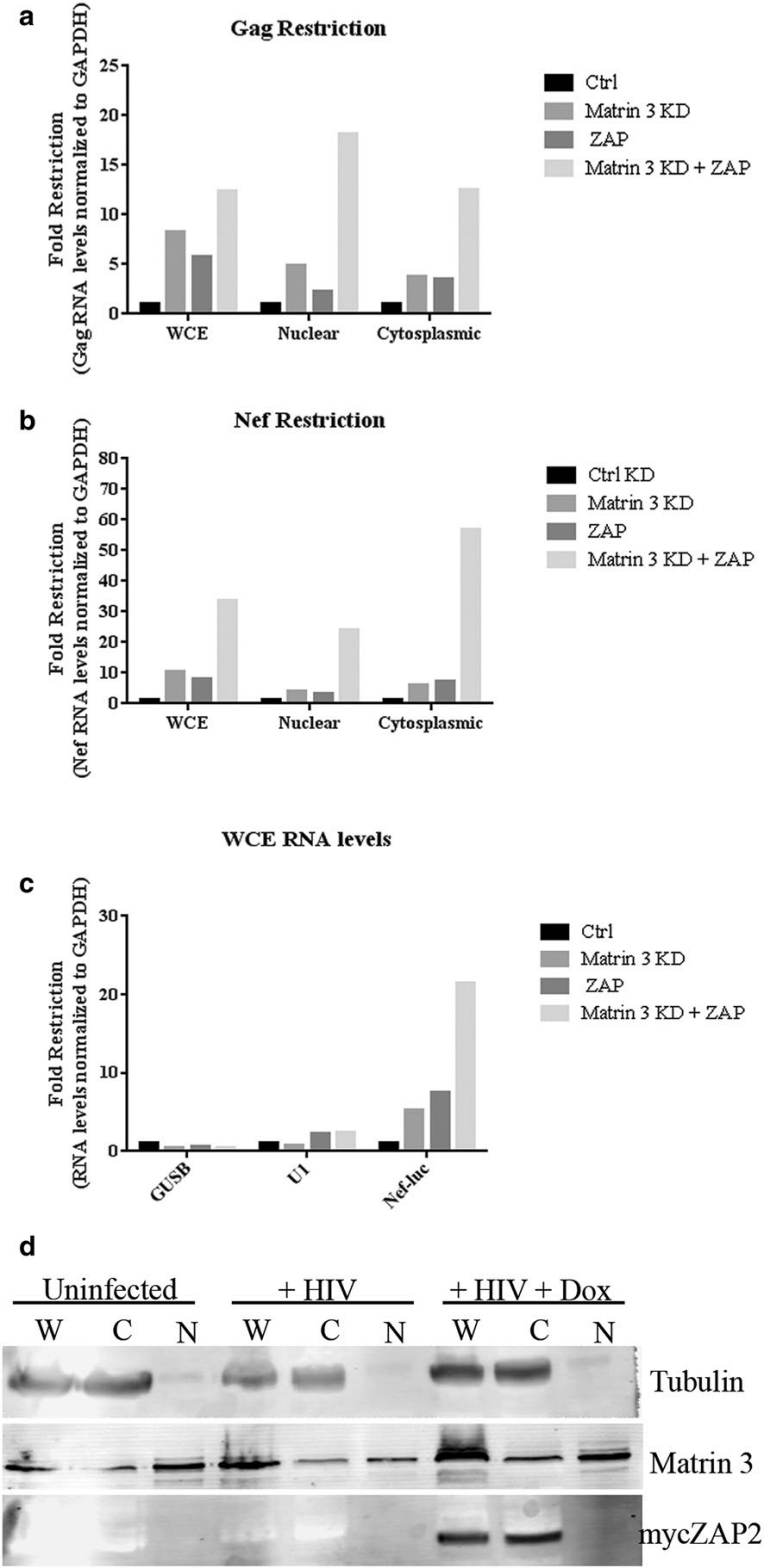
Matrin 3 knockdown improves the ZAP-induced degradation of HIV-1 transcripts. Greater unspliced and multiply-spliced RNA degradation with simultaneous ZAP induction and Matrin 3 knockdown. 293TrexhZAP2 cells were transfected with control siRNA (Ctrl) or siRNA against Matrin 3 (Matrin 3 KD) for two consecutive days. The following day cells were infected with HIV-luc then treated with doxycycline treatment at 200 ng/ml (ZAP) or without. Two days postinfection, RNA was isolated from whole cell extracts (WCE), as well as from nuclear and cytoplasmic compartments. QRT-PCR analysis was performed on HIV-1 RNA using specific primers against **a** unspliced transcripts (Gag) and **b** multiply-spliced transcripts (Nef). Relative levels of these transcripts normalized to GAPDH levels are presented. Fold restriction was calculated from dividing Ctrl siRNA values by the results of each treatment indicated within the WCE, nuclear, or cytoplasmic compartment measured. Data presented are from a representative experiment done in triplicate. Similar results were obtained for three independent experiments. **c** QRT-PCR analysis of WCE RNA using specific primers against cellular transcripts, beta-glucuronidase (GUSB), Small Nuclear Ribonucleoprotein 70 kDa (U1), or HIV-1 Nef-luc. Fold restriction was calculated as above. Similar results were obtained for two independent experiments. **d** An immunoblot of the cytoplasmic (Tubulin) and nuclear fractions (Matrin 3). Compartmentalization of myc-ZAP2 is also shown.

Both Matrin 3 and ZAP are known to have effects on HIV-1 mRNA levels that are specific to particular splicing classes of mRNAs. Matrin 3 is necessary for the accumulation of Rev-dependent unspliced and singly-spliced transcripts in the cytoplasm and has been documented to act as a cofactor of Rev [6, 16]. ZAP targets most strongly multiply-spliced transcripts for degradation, and has smaller effects on unspliced and singly-spliced mRNAs [27]. To investigate which HIV-1 RNA transcripts were the target of the augmented ZAP restriction upon Matrin 3 knockdown, we used qRTPCR to monitor RNA levels upon ZAP expression, Matrin 3 knockdown, or both. Following RNA extraction of HIV-luc infected 293TrexhZAP2 cells, we quantified unspliced (Figure 4a) and multiply-spliced transcripts (Figure 4b). In whole cell extracts (WCE), unspliced mRNA was reduced by approximately 8-fold with Matrin 3 KD and 6-fold with ZAP overexpression, and by approximately 12-fold with both (Figure 4a). Multiply-spliced RNA levels (Figure 4b) were reduced approximately 11-fold with Matrin 3 KD or 8-fold with ZAP overexpression. Together, however, these conditions led to a considerably greater 34-fold reduction in multiply-spliced mRNAs. The level of HIV-1 fold reduction in multiply-spliced (Nef-luc) RNA levels with both Matrin 3 KD and ZAP overexpression was similar to that observed for Nef-luc protein expression in luciferase experiments (Figure 2a), strongly suggesting that Matrin 3 inhibits the ZAP-mediated degradation of HIV-1 RNAs.

Previously it was found that ZAP specifically eliminated MoMuLV RNA in the cytoplasm, and not the nucleus [18]. Whether a similar compartmentalization for RNA elimination for other ZAP-sensitive viruses is yet to be defined. To determine if the observed reduction in HIV-1 transcript levels were restricted to a subcellular compartment, mRNA levels were measured in nuclear and cytoplasmic fractions. Unspliced mRNA in nuclear fractions was reduced in Matrin 3 KD cells or with ZAP overexpression, and these conditions together produced a larger 18-fold reduction in mRNA levels. Comparable reductions in unspliced mRNAs were observed in the cytoplasmic fraction, with Matrin 3 KD and ZAP overexpression together resulting in a 13-fold reduction (Figure 4a). Matrin 3 KD or ZAP overexpression individually led to similar reductions of the multiply-spliced mRNAs in both the nucleus and cytoplasmic fractions, while together these conditions lead to a remarkable 24-fold reduction and 57-fold reduction in RNA levels in the nuclear and cytoplasmic compartments respectively (Figure 4b). Cellular genes beta-glucuronidase (GUSB) and small Nuclear Ribonucleoprotein 70 kDa (U1) were not substantially affected under any conditions, suggesting that Matrin 3 KD and ZAP overexpression together was not causing global reductions in RNA levels (Figure 4c.) To test for the effectiveness of the fractionation, the distribution of tubulin was examined by Western blot. Tubulin was located chiefly in the cytoplasmic fraction, showing that there was efficient separation of these subcellular compartments (Figure 4d). Visualization of myc-ZAP2 following fractionation showed that at steady state ZAP localized predominantly to the cytoplasmic fractions (Figure 4d) as noted in past studies of the rat homolog of ZAP [35]. Western blots for Matrin 3 showed predominant localization to the nucleus, but with some localization to the cytoplasm. These localizations are consistent with the observations that the changes in RNA levels are happening in both cytoplasm and nucleus. Quantification of Matrin 3 RNA showed no effect of HIV-1 infection or ZAP overexpression on Matrin 3 RNA levels (data not shown).

Taken together these results indicate that although knockdown of Matrin 3 or overexpression of ZAP alone can decrease HIV-1 mRNAs levels, when ZAP is overexpressed in the context of Matrin 3 KD a drastically enhanced reduction of both unspliced and multiply-spliced HIV-1 transcripts occurs. This suggests that Matrin 3 is negatively regulating ZAP-mediated HIV-1 restriction and may be serving in a HIV-1 RNA protective function. Furthermore, this effect is noted in both nuclear and cytoplasmic compartments, and most prominently for multiply-spliced transcripts.

## Discussion

Matrin 3 is a host cell protein involved in the replication cycle of multiple viruses [6, 8, 16]. In HIV-1 infection, Matrin 3 was shown to act as a Rev cofactor and promotes the accumulation of HIV-1 unspliced and singly-spliced transcripts in the cytoplasm [6, 16]. Here we demonstrate that Matrin 3 associates with three key proteins of the ZAP degradation complex and that silencing of Matrin 3 expression causes a remarkable increase in ZAP’s restriction of retroviral transcripts. Thus our data show that Matrin 3 serves as a negative regulator of ZAP.

Intriguingly, many of the proteins identified by mass spectrometry as binding to Matrin 3 (Table 1) were RNA binding proteins involved in mRNA processing and transport. DEAD-box proteins such as DDX17, many of which are putative helicases [36], function in various RNA-mediated processes. Another family of binding proteins, the HNRNPs, are highly abundant nuclear RNA-binding proteins that are amongst the core proteins that bind and package recently transcribed nascent nuclear pre-mRNAs into ribonucleoprotein complexes, and also play important roles in constitutive and alternative splicing. HNRNPs are involved in both DNA and RNA metabolism, often through their recruitment of regulatory proteins involved in these processes. Additionally HNRNPs may remain bound to pre-mRNAs as chaperones or regulators through their RNA biogenesis and later maturation in the cytoplasm [37, 38].

A first indication that Matrin 3 held a role in the ZAP degradation complex was its binding to DDX17 and EXOSC3. The mass spectrophotometric analysis of Matrin 3-associated proteins repeatedly identified DDX17 and EXOSC3, both integral components of the ZAP degradation machinery. Matrin 3 interacted with these proteins in a largely RNA-dependent manner at endogenous levels (Figure 1a, b). Additionally myc-tagged ZAP2 bound to Matrin 3 in an RNA-dependent (i.e. RNase-sensitive) manner. As RNAs are involved in bridging the proteins, one model for the mechanism of Matrin 3’s regulation of ZAP’s antiviral activity may be through competition for similar sites on viral RNAs. Its interactions with proteins of the complex may reveal a dynamic competition for sites on targeted cellular and/or viral RNAs.

We found that loss of Matrin 3 expression alone had significant effects on HIV-1 RNA levels. Matrin 3 silencing caused HIV-1 transcripts levels to drop well below those of controls (Figure 4a, b). A number of RNA binding proteins (RBPs) important to HIV-1 RNA processing and export, such as DDX3, DDX5, DDX17, and PTBP1 [26, 27, 39–43] were identified in our screen (Table 1), and thus it is plausible that Matrin 3 is providing a scaffold for proper RBP assembly on HIV-1 RNA transcripts. Many of these proteins were also identified as Matrin 3-associating proteins in a previous mass spectrometry analysis using exogenously expressed Matrin 3 [7]. Moreover, a yeast two hybrid analysis identified SLTM, DDX5 [9] and PTBP1 [16] as Matrin 3-associating proteins. HNRNPU and Matrin 3 have been proposed to serve as a scaffold at sites of DNA replication and transcription due to their colocalization within dynamic nuclear microenvironments [14]. Hence, these interactions support the idea that Matrin 3 is acting in complex with key RNA-binding proteins to regulate various processes to ensure the proper maturation, transport and transcript levels of both host and viral transcripts.

The overexpression of ZAP in the context of Matrin 3 suppression caused the most marked decrease in HIV-1 transcript levels, far exceeding the decrease seen in either condition alone. ZAP as reported previously substantially lowered levels of HIV-1 transcripts especially multiply spliced transcripts, but when endogenous levels of Matrin 3 were lowered, the magnitude of the reduction was increased, and was extended to both unspliced and multiply spliced HIV-1 transcripts. Furthermore our study suggests that Matrin 3’s negative regulation of ZAP may extend to the restriction of other ZAP-sensitive viruses (Figures 2b, 3b). We theorize that at endogenous levels, Matrin 3 provides a baseline inhibition of ZAP-mediated degradation that for HIV-1 may begin in the nucleus and continue into the cytoplasm. Alternatively, this negative regulation may reflect Matrin 3 involvement in exclusive restriction pathways in these subcellular compartments. Removal of Matrin 3 augments and broadens ZAP’s ability to inhibit HIV-1 infection—exposes the RNAs to attack by ZAP—and in effect leads to a more potent retroviral restriction factor.

Matrin 3 is known to interact with Rev, and operates as its cofactor to aid the expression of unspliced and singly-spliced transcripts but not Rev-independent multiply-spliced transcripts. Rev functions to inhibit the splicing and/or nuclear retention brought on by cis-repressor sequences in unspliced and singly-spliced transcripts [44]. However our findings indicate that Matrin 3’s effect on ZAP activity is likely a Rev-independent process, as a similar trend was seen with Rev-independent reporters, and with MoMuLV (Figure 2b) which does not express Rev. Loss of Matrin 3 expression may lead unspliced and multiply-spliced transcripts to be retained in the nucleus and more vulnerable to nuclear and/or cytoplasmic RNA degradation. It is known that RBPs recruited during transcription can compete and therefore protect nascent RNAs from nuclear RNA surveillance machinery [38]. Hence, Matrin 3 may be functioning as an RBP in a RNA protective role. This function expands on the role of Matrin 3 not only as a Rev cofactor, important for the accumulation of unspliced and singly-spliced transcripts in the cytoplasm [6, 16], but also in the regulation of ZAP-mediated restriction of HIV-1 gene expression.

Transcription, splicing, localization and decay are often all interconnected steps in the course of RNA maturation [38]. The regulatory RBPs functioning in these roles may bind to nascent RNA and during their synthesis “configure” them with the necessary RBP’s and other host proteins needed for the proper packaging, transport and processing of the transcript. Matrin 3 has been proposed to act as a scaffold at active sites of transcription [14], and to interact with key proteins involved in RNA metabolism. The absence of Matrin 3 may have direct effects on the assembly of necessary proteins for both cellular and viral RNA maturation, and improper packaging of transcripts may have negative effects on their overall maturation and stability. Further evidence for Matrin 3 acting to provide RNA stability for cellular and viral transcripts has been supported by our findings here and in previous studies where it was established to bind and control the stability of host cell RNA [7]. Matrin 3 has independently been suggested to function as an RBP positively regulating mRNA stability [45]. Other nuclear matrix proteins which are also RNA binding proteins involved in RNA metabolism display a commonality as to their effects on ZAP-mediated retroviral restriction, as seen for HNRNPU and SFPQ (Figure 3). Hence our studies reveal that this negative regulation of ZAP restriction may be a shared function of other nuclear matrix RBPs.

Future studies will be directed at finding in more detail how Matrin 3 is involved in regulating ZAP-mediated restriction of retroviruses. As Matrin 3 has been found to bind to both host and viral RNAs, Matrin 3 may compete with ZAP for access to ZAP recognition motifs on HIV-1 RNAs, and thus suppression of Matrin 3 may leave both unspliced and multiply spliced transcripts further accessible to ZAP degradation machinery. ZAP molecules interact [46] and form dimers on ZAP-sensitive RNAs [47], and hence another possibility is that Matrin 3 is inhibiting ZAP from properly dimerizing on HIV-1 RNAs. Binding of Matrin 3 to DDX17, EXOSC3, and ZAP in uninfected cells may reflect a role for Matrin 3 in alternate host RNA processes, but during infection Matrin 3 may impede ZAP from interacting with these proteins which are needed for optimal RNA degradation activity. Our study offers new insights into a function for Matrin 3 in the regulation of an HIV-1 restriction factor and expands on the potential for Matrin 3 in controlling HIV-1 gene expression.

## Conclusions

Matrin 3, a matrix protein involved in multiple nuclear process, is important for the accumulation of unspliced and singly-spliced HIV-1 transcripts. ZAP is a retroviral restriction factor that leads to the degradation of largely multiply-spliced HIV-1 transcripts. Here we demonstrate that Matrin 3 interacts with key proteins of the ZAP-degradation complex, and suppression of Matrin 3 drastically improves ZAPs ability to block HIV-1 infection, with similar findings for MoMuLV. Silencing of Matrin 3 also broadens ZAPs ability to degrade multiple classes of HIV-1 RNA. Our result supports the concept that Matrin 3 impedes ZAP-mediated retroviral RNA degradation. As Matrin 3 is a highly conserved protein, this Matrin 3 regulation of ZAP antiviral activity may extend to other ZAP-sensitive viruses.

## Methods

### Cell culture, transfections, plasmids, virus packaging and infections

293TrexhZAP2 cells have been previously described [27]. Briefly 293Trex cells (Invitrogen) were stably transfected with a tetracycline-inducible myc-tagged hZAP-v2, an alternatively spliced form of ZAP that differs at the C terminus. CHME3, a human microglial cell line kindly provided by Dr. Mojgan Naghavi, Northwestern University were maintained in DMEM (Dulbecco’s Modified Eagle Medium) supplemented with 10% fetal bovine serum, Sodium Pyruvate, l-Glutamine, and Penicillin–Streptomycin (Gibco).

Matrin 3 tagged at the C-terminus with MycDDK was generated from a commercial plasmid of matrin 3 gene (pCMV SPORT 6, Invitrogen) that was PCR amplified with oligonucleotides Matrin 3F (5′-GAGGCGATCGCATGTCCAAGTCATTCCA-3′) and Matrin 3R (5′-GCGACGCGTAGTTTCCTTCTTCTGTCT-3′) using KOD Hot Start DNA Polymerase (Novagen). The PCR fragment was digested with Mlu1and AsiS1 and inserted into a MycDDK vector (Origene). MycDDK empty vector was generated by digesting a MycDDK tagged ORF clone with Xho1 and Sal1 followed by religation of the vector fragment. Plasmids were transfected into 293TrexhZAP2 cells with Lipofectamine 2000 (Invitrogen) according to the manufacturer’s protocol.

To produce vesicular stomatitis virus envelope glycoprotein (VSV-G) pseudotyped HIV-1 virus, HEK293T cells were cotransfected with plasmids pMDG (plasmid expressing VSV-G) and pNL4.3Env^−^Luc^+^ (based on HIV-1 provirus pNL4.3 with Firefly luciferase gene inserted into Nef gene and two frameshifts rendering the clone Env^−^ and Vpr^−^) by Calcium Phosphate transfection method. VSV-G pseudotyped Moloney Murine Leukemia Virus (MoMuLV) were produced by cotransfection with pMDG, pCMVintron (plasmid encoding NB MoMuLV gag-pol) and pFB-luc (Stratagene) (transducing vector expressing Firefly luciferase). VSV-G HR’CMV-luc in which a large portion of the HIV-1 genome is deleted and luciferase activity is driven by a CMV promoter is described in Ref. [27]. Cells were cotransfected and media was changed the following day. Viral supernatants were harvested from cells 48 h post-transfection and passed through a 0.45 µm pore filter. For infections, viral supernatants were overlaid on cells for 2 h, then replaced with media.

To measure firefly luciferase reporter activity, luciferase assay reagent (Luciferase assay system, Promega) was added to lysates and monitored using the POLARstar Omega multi-mode microplate reader (BMG Labtech). Protein levels of extracts were normalized by Bradford assay prior to measurement.

### Antibodies

Mouse α-DDX17 (Santa Cruz, Santa Cruz, CA, USA), mouse α -Exosc3 (Santa Cruz), mouse α-c-Myc (Santa Cruz), mouse α-DDK (Origene, Rockville, MD, USA), mouse α-α-Tubulin (Sigma-Aldrich, St. Louis, MO, USA), mouse α-GAPDH (EMD Chemicals, Inc., San Diego, CA, USA), and secondary IRDye 680LT donkey polyclonal α-mouse IgG (H + L) (LI-COR, Lincoln, NE, USA) and donkey α-rabbit IgG (H&L) IRDye 800 (Rockland, Gilbertsville, PA, USA) were used in immunoblot analysis. Rabbit α-Matrin 3 (Bethyl Laboratories, Inc., Montgomery, TX, USA) was used in immunoblot analysis and with Normal Rabbit IgG polyclonal antibody (Millipore, Temecula, CA, USA) in coimmunoprecipitation studies.

### Immunoblot analysis and Coimmunoprecipitations

Cells were washed in cold PBS, then harvested with lysis buffer (20 mM Tris–HCl pH 7.5, 150 mM NaCl, 1% NP40) and sonicated on ice in the presence of protease and phosphatase inhibitors (Roche). RNAse A (50 µg/ml) was added to lysates and incubated at RT for 1 h. Lysates were then centrifuged for 30 min at 13,000 rpm and a sample for whole cell extract (WCE) was collected. The soluble fraction was then subjected to immunoprecipitation.

Dynabeads Protein G (Invitrogen) were washed and then bound to 1 µg of control rabbit IgG antibody or rabbit α-Matrin3 with rotation for 1.5 h at 4°C in lysis buffer. These were then washed four times before adding the beads to lysates. Antibody-bound beads were incubated with lysates overnight at 4°C, then washed four times with lysis buffer for a total of 30 min. SDS sample buffer was added to beads. Samples were then boiled and separated from the beads and collected for further analysis.

### Mass spectrometry

CHME3 and CHME3 cells infected with HIV-luc were harvested 2 days postinfection. These were lysed in lysis buffer (20 mM Tris–HCl pH 7.5, 150 mM NaCl, 1% NP40) and sonicated on ice in the presence of protease and phosphatase inhibitors. Lysates were then centrifuged for 30 min at 13,000 rpm and the soluble fraction was then subjected to immunoprecipitation. To minimize the elution of immunoglobulins following immunoprecipitation, Dynabeads Protein G (Invitrogen) were crosslinked with 10 µg of control rabbit IgG antibody or rabbit α-Matrin3. Antibody bound beads were washed in citrate phosphate buffer, followed by a wash in sodium borate. Beads were then resuspended in 20 mM DMP in sodium borate for 30 min at 20°C. This was followed by a wash with TBST (TBS with 0.1% Tween-20). Subsequently the beads were incubated with lysates at 4°C, and washed with 4 times with lysis buffer for a total of 30 min. SDS sample buffer without beta mercaptoethanol was added to the beads and the supernatant was separated from the beads. Βeta mercaptoethanol was then added to the supernatants and boiled. Samples were then briefly run on an SDS- PAGE gel. Samples were excised from the resolving gel and sent for LC–MS/MS analysis at the Protein Core facility of the Herbert Irving Comprehensive Cancer Center at Columbia University. LC–MS/MS analysis was done on a Waters Ultima Q-Tof hybrid quadrupole/time-of-flight mass spectrometer with a nanoelectrospray source. Raw data files were processed using the MassLynx ProteinLynx software and .pkl files were submitted for searching at www.matrixscience.com using the Mascot algorithm.

### siRNA knockdown

siRNA against Matrin 3 (Catalog #HSS114730, Invitrogen, Carlsbad, CA, USA), SFPQ (Catalog #HSS109642), HNRNPU (Catalog #HSS104917), Lamin A (Catalog #HSS106095), and negative control, Stealth RNAi siRNA Negative Control Med GC Duplex #2 (Invitrogen, Carlsbad, CA, USA) were used. 293TrexhZAP2 cells were transfected with siRNAs using Lipofectamine RNAiMAX (Invitrogen) according to manufacturer’s instructions. Transfections were performed for two consecutive days before infection the following day.

### Statistical analysis

The mean values ± SD from were calculated from at least three independent experiments unless otherwise indicated. Unpaired *t* test were performed using GraphPad Prism version 6.04 Software. *P* values ≤0.05 were considered significant.

### Quantitative real-time RT-PCR (qRT-PCR) and nuclear/cytoplasmic fractionation

Cells were separated into nuclear and cytoplasmic fractions (adapted from [48]) prior to qRT-PCR analysis. Briefly cells were washed with PBS, followed by resuspension in lysis buffer [10 mM Tris 8.0, 1.5 mM MgCl_2_, 140 mM NaCl, 10 mM EDTA, 0.5% NP40 and 0.3 U/ml RNaseOUT (Invitrogen)]. A sample was obtained for whole cell extract RNA. Extracts were then centrifuged twice at 3,000 rpm for 5 min at 4°C, and the supernatants containing the cytoplasmic fraction were harvested. The remaining nuclear pellets were washed twice with lysis buffer, and passed through a 20-gauge needle.

RNA was isolated from 293TrexhZAP2 WCE, nuclear and cytoplasmic fractions using TRIzol Reagent (Ambion Invitrogen). To remove potential DNA contamination, RNAs were DNAse treated using a TURBO DNA-free kit (Ambion Invitrogen). 2 µg of RNA was then reverse transcribed with High-Capacity cDNA Reverse Transcription kit (Applied Biosystem, Foster City, CA, USA) according to manufacturer’s instructions. RNA levels were then measured using FastStart Universal SYBR Green Master (Rox). Here 3 μl of diluted cDNA, diluted primer and SYBR green master mix were added to a total 20 μl reaction and measured in the 7500 Fast Real-Time PCR system (Applied Biosytems) using the following PCR program: (1) 50°C 2 min, 1 cycle (2) 95°C 10 min, 1 cycle (3) 95°C 15 s → 60°C 1 min, 40 cycles. Target gene mRNA expression was normalized to GAPDH expression.

The sequence of the primers used in qRT-PCR are as follows:

qGAG FP 5′-GTGTGGAAAATCTCTAGCAGTGG-3′

qGAG RP 5′-CGCTCTCGCACCCATCTC-3′

qNef-luc FP 5′-ACAGTCAGACTCATCAAGCTTCTCT-3′

qNef-luc RP 5′-CGGGTCCCCTCGGGATT-3′

qMatr3 FP 5′-GCGCCTTTCTTGCTCGCTCC-3′

qMatr3 RP 5′-ACCAGCAGACAACTCTCCGCC-3′

qGAPDH FP 5′-TTTTGCGTCGCCAGCCGAG-3′

qGAPDH RP 5′-TGACCAGGCGCCCAATACGAC-3′

qU1 FP 5′-AGGGCGAGGCTTATCCATT-3′

qU1 RP 5′-GCAGTCGAGTTTCCCACATT-3′

qGUSB FP 5′-CACCAGGGACCATCCAATACC-3′

qGUSB RP 5′-GCAGTCCAGCGTAGTTGAAAAA-3′

## References

[1] Belgrader P, Dey R, Berezney R (1991). Molecular cloning of matrin 3. A 125-kilodalton protein of the nuclear matrix contains an extensive acidic domain. J Biol Chem.

[2] Berezney R, Coffey DS (1974). Identification of a nuclear protein matrix. Biochem Biophys Res Commun.

[3] Nakayasu H, Berezney R (1991). Nuclear matrins: identification of the major nuclear matrix proteins. Proc Natl Acad Sci USA.

[4] Engelke R, Riede J, Hegermann J, Wuerch A, Eimer S, Dengjel J (2014). The quantitative nuclear matrix proteome as a biochemical snapshot of nuclear organization. J Proteome Res.

[5] Albrethsen J, Knol JC, Jimenez CR (2009). Unravelling the nuclear matrix proteome. J Proteomics.

[6] Kula A, Guerra J, Knezevich A, Kleva D, Myers MP, Marcello A (2011). Characterization of the HIV-1 RNA associated proteome identifies Matrin 3 as a nuclear cofactor of Rev function. Retrovirology.

[7] Salton M, Elkon R, Borodina T, Davydov A, Yaspo ML, Halperin E (2011). Matrin 3 binds and stabilizes mRNA. PLoS One.

[8] Erazo A, Yee MB, Banfield BW, Kinchington PR (2011). The alphaherpesvirus US3/ORF66 protein kinases direct phosphorylation of the nuclear matrix protein matrin 3. J Virol.

[9] Zeitz MJ, Malyavantham KS, Seifert B, Berezney R (2009). Matrin 3: chromosomal distribution and protein interactions. J Cell Biochem.

[10] Giordano G, Sanchez-Perez AM, Montoliu C, Berezney R, Malyavantham K, Costa LG (2005). Activation of NMDA receptors induces protein kinase A-mediated phosphorylation and degradation of matrin 3. Blocking these effects prevents NMDA-induced neuronal death. J Neurochem.

[11] Zhang Z, Carmichael GG (2001). The fate of dsRNA in the nucleus: a p54(nrb)-containing complex mediates the nuclear retention of promiscuously A-to-I edited RNAs. Cell.

[12] Salton M, Lerenthal Y, Wang SY, Chen DJ, Shiloh Y (2010). Involvement of Matrin 3 and SFPQ/NONO in the DNA damage response. Cell Cycle.

[13] Ma H, Siegel AJ, Berezney R (1999). Association of chromosome territories with the nuclear matrix. Disruption of human chromosome territories correlates with the release of a subset of nuclear matrix proteins. J Cell Biol.

[14] Malyavantham KS, Bhattacharya S, Barbeitos M, Mukherjee L, Xu J, Fackelmayer FO (2008). Identifying functional neighborhoods within the cell nucleus: proximity analysis of early S-phase replicating chromatin domains to sites of transcription, RNA polymerase II, HP1gamma, matrin 3 and SAF-A. J Cell Biochem.

[15] Johnson JO, Pioro EP, Boehringer A, Chia R, Feit H, Renton AE (2014). Mutations in the Matrin 3 gene cause familial amyotrophic lateral sclerosis. Nat Neurosci.

[16] Yedavalli VS, Jeang KT (2011). Matrin 3 is a co-factor for HIV-1 Rev in regulating post-transcriptional viral gene expression. Retrovirology.

[17] Stoltzfus CM (2009). Regulation of HIV-1 alternative RNA splicing and its role in virus replication, chapter 1. Adv Virus Res.

[18] Gao G, Guo X, Goff SP (2002). Inhibition of retroviral RNA production by ZAP, a CCCH-type zinc finger protein. Science.

[19] Bick MJ, Carroll JW, Gao G, Goff SP, Rice CM, MacDonald MR (2003). Expression of the zinc-finger antiviral protein inhibits alphavirus replication. J Virol.

[20] Muller S, Moller P, Bick MJ, Wurr S, Becker S, Gunther S (2007). Inhibition of filovirus replication by the zinc finger antiviral protein. J Virol.

[21] Mao R, Nie H, Cai D, Zhang J, Liu H, Yan R (2013). Inhibition of hepatitis B virus replication by the host zinc finger antiviral protein. PLoS Pathog.

[22] Xuan Y, Liu L, Shen S, Deng H, Gao G (2012). Zinc finger antiviral protein inhibits murine gammaherpesvirus 68 M2 expression and regulates viral latency in cultured cells. J Virol.

[23] Guo X, Carroll JW, Macdonald MR, Goff SP, Gao G (2004). The zinc finger antiviral protein directly binds to specific viral mRNAs through the CCCH zinc finger motifs. J Virol.

[24] Guo X, Ma J, Sun J, Gao G (2007). The zinc-finger antiviral protein recruits the RNA processing exosome to degrade the target mRNA. Proc Natl Acad Sci USA.

[25] Zhu Y, Gao G (2008). ZAP-mediated mRNA degradation. RNA Biol.

[26] Chen G, Guo X, Lv F, Xu Y, Gao G (2008). p72 DEAD box RNA helicase is required for optimal function of the zinc-finger antiviral protein. Proc Natl Acad Sci USA.

[27] Zhu Y, Chen G, Lv F, Wang X, Ji X, Xu Y (2011). Zinc-finger antiviral protein inhibits HIV-1 infection by selectively targeting multiply spliced viral mRNAs for degradation. Proc Natl Acad Sci USA.

[28] Zhu Y, Wang X, Goff SP, Gao G (2012). Translational repression precedes and is required for ZAP-mediated mRNA decay. EMBO J.

[29] Janabi N, Peudenier S, Heron B, Ng KH, Tardieu M (1995). Establishment of human microglial cell lines after transfection of primary cultures of embryonic microglial cells with the SV40 large T antigen. Neurosci Lett.

[30] Gonzalez-Scarano F, Martin-Garcia J (2005). The neuropathogenesis of AIDS. Nat Rev Immunol.

[31] Brouwer R, Allmang C, Raijmakers R, van Aarssen Y, Egberts WV, Petfalski E (2001). Three novel components of the human exosome. J Biol Chem.

[32] Meissner M, Dechat T, Gerner C, Grimm R, Foisner R, Sauermann G (2000). Differential nuclear localization and nuclear matrix association of the splicing factors PSF and PTB. J Cell Biochem.

[33] Mattern KA, van Goethem RE, de Jong L, van Driel R (1997). Major internal nuclear matrix proteins are common to different human cell types. J Cell Biochem.

[34] Skowronska-Krawczyk D, Ma Q, Schwartz M, Scully K, Li W, Liu Z (2014). Required enhancer-matrin-3 network interactions for a homeodomain transcription program. Nature.

[35] Liu L, Chen G, Ji X, Gao G (2004). ZAP is a CRM1-dependent nucleocytoplasmic shuttling protein. Biochem Biophys Res Commun.

[36] Jarmoskaite I, Russell R (2011). DEAD-box proteins as RNA helicases and chaperones. Wiley Interdiscip Rev RNA.

[37] Dreyfuss G, Kim VN, Kataoka N (2002). Messenger-RNA-binding proteins and the messages they carry. Nat Rev Mol Cell Biol.

[38] Muller-McNicoll M, Neugebauer KM (2013). How cells get the message: dynamic assembly and function of mRNA-protein complexes. Nat Rev Genet.

[39] Yedavalli VS, Neuveut C, Chi YH, Kleiman L, Jeang KT (2004). Requirement of DDX3 DEAD box RNA helicase for HIV-1 Rev-RRE export function. Cell.

[40] Naji S, Ambrus G, Cimermancic P, Reyes JR, Johnson JR, Filbrandt R (2012). Host cell interactome of HIV-1 Rev includes RNA helicases involved in multiple facets of virus production. Mol Cell Proteomics.

[41] Zhou X, Luo J, Mills L, Wu S, Pan T, Geng G (2013). DDX5 facilitates HIV-1 replication as a cellular co-factor of Rev. PLoS One.

[42] Lorgeoux RP, Pan Q, Le Duff Y, Liang C (2013). DDX17 promotes the production of infectious HIV-1 particles through modulating viral RNA packaging and translation frameshift. Virology.

[43] Lassen KG, Ramyar KX, Bailey JR, Zhou Y, Siliciano RF (2006). Nuclear retention of multiply spliced HIV-1 RNA in resting CD4+ T cells. PLoS Pathog.

[44] Dayton AI (2011). Matrin 3 and HIV Rev regulation of mRNA. Retrovirology.

[45] Ray D, Kazan H, Cook KB, Weirauch MT, Najafabadi HS, Li X (2013). A compendium of RNA-binding motifs for decoding gene regulation. Nature.

[46] Law LM, Albin OR, Carroll JW, Jones CT, Rice CM, Macdonald MR (2010). Identification of a dominant negative inhibitor of human zinc finger antiviral protein reveals a functional endogenous pool and critical homotypic interactions. J Virol.

[47] Chen S, Xu Y, Zhang K, Wang X, Sun J, Gao G (2012). Structure of N-terminal domain of ZAP indicates how a zinc-finger protein recognizes complex RNA. Nat Struct Mol Biol.

[48] Weil D, Boutain S, Audibert A, Dautry F (2000). Mature mRNAs accumulated in the nucleus are neither the molecules in transit to the cytoplasm nor constitute a stockpile for gene expression. RNA.

